# Contribution of reactive oxygen species to the anticancer activity of aminoalkanol derivatives of xanthone

**DOI:** 10.1007/s10637-017-0537-x

**Published:** 2017-11-08

**Authors:** Daniel Sypniewski, Natalia Szkaradek, Tomasz Loch, Anna M. Waszkielewicz, Agnieszka Gunia-Krzyżak, Daria Matczyńska, Dagna Sołtysik, Henryk Marona, Ilona Bednarek

**Affiliations:** 1 Department of Biotechnology and Genetic Engineering, School of Pharmacy with the Division of Laboratory Medicine in Sosnowiec, Medical University of Silesia, Katowice, Poland; 2 Department of Bioorganic Chemistry, Chair of Organic Chemistry, Faculty of Pharmacy, Jagiellonian University Medical College, Krakow, Poland

**Keywords:** Xanthone, Cancer, Reactive oxygen species (ROS), Antioxidant enzymes, Senescence

## Abstract

Reactive oxygen species (ROS) are critically involved in the action of anticancer agents. In this study, we investigated the role of ROS in the anticancer mechanism of new aminoalkanol derivatives of xanthone. Most xanthones used in the study displayed significant pro-oxidant effects similar to those of gambogic acid, one of the most active anticancer xanthones. The pro-oxidant activity of our xanthones was shown both directly (by determination of ROS induction, effects on the levels of intracellular antioxidants, and expression of antioxidant enzymes) and indirectly by demonstrating that the overexpression of manganese superoxide dismutase decreases ROS-mediated cell senescence. We also observed that mitochondrial dysfunction and cellular apoptosis enhancement correlated with xanthone-induced oxidative stress. Finally, we showed that the use of the antioxidant N-acetyl-L-cysteine partly reversed these effects of aminoalkanol xanthones. Our results demonstrated that novel aminoalkanol xanthones mediated their anticancer activity primarily through ROS elevation and enhanced oxidative stress, which led to mitochondrial cell death stimulation; this mechanism was similar to the activity of gambogic acid.

## Introduction

Reactive oxygen species (ROS) are molecules derived from intracellular oxygen metabolism or from the extracellular environment. Excessive ROS levels generate oxidative stress, where oxidation of macromolecules causes significant damage to cellular organelles and genes and subsequently leads to apoptotic cell death. Multiple disorders have been associated with oxidative stress, and oxidative stress plays a crucial role in the progression of cancer as well as cancer treatment [1]. Redox homeostasis is maintained by the intracellular antioxidant system, which consists of compounds such as reduced glutathione, α-tocopherol or ascorbic acid that protect cellular macromolecules from oxidative damage. However, the most powerful element of the cellular defense system is based on antioxidant enzymes: superoxide dismutase (SOD), catalase (CAT), and glutathione peroxidase (GPX) [2]. As some biological systems evolved with environments rich in ROS, ROS became an important part of cellular signaling pathways. Most significantly, ROS play a key role in the regulation of apoptosis and proliferation [1]. While slight increases in ROS may promote cell proliferation and differentiation [3], excess ROS levels lead to oxidative damage and induction of apoptosis [4].

Cancer cells activate mechanisms to avoid ROS-mediated apoptosis and to enhance ROS-mediated proliferation and development. One such a mechanism, the “SOD paradox”, is based on the observation that MnSOD (mitochondrial form of SOD) expression decreases during neoplastic transformation, leading to accumulation of superoxide anion, which in turn stimulates cell growth via specific transcription factors [1]. However, as the tumor cells develop, MnSOD expression significantly increases. This is accompanied by a decrease in CAT and GPX expression, and the overall ROS levels constantly increase [5]. As a result, cancer cells are under permanent oxidative stress [2, 3]. Another important issue is the participation of ROS in the mechanisms of anticancer therapies. ROS mediate a significant proportion of the anticancer effects of agents used in chemo-, radio-, and photodynamic therapies [1, 6]. Oxidative stress is particularly enhanced by alkylating agents and topoisomerase II inhibitors, but other drugs (approximately 40% of chemotherapeutic agents) also exhibit oxidative activity. Application of ROS-generating agents during anticancer therapy leads to a situation where drug-induced oxidative stress superimposes on the intrinsic stress, which results in the preferential death of tumor cells or inhibition of their proliferation. Therefore, a significant mechanism of anticancer therapies is based on either accumulation of ROS or inhibition of ROS neutralization. Since this “oxidation therapy” is a double-edged sword, some researchers have proposed the inclusion of antioxidants as therapeutic adjuvants [7]. While the use of antioxidants as chemopreventive agents is well established, their application in cancer therapy is controversial. The concomitant use of antioxidants may be beneficial not only due to their protective role in normal cells but also due to their positive impact on chemotherapeutic efficiency. However, when high amounts of ROS are limited through the application of antioxidants, elimination of cancer cells by ROS may also be blocked, mostly via inhibition of ROS-mediated apoptosis [2].

Xanthone (9*H*–xanthen-9-one) is an oxygenated, heterocyclic compound. The large group of compounds based on the xanthonic core are termed “xanthone derivatives” or simply “xanthones”. These compounds are natural metabolites found primarily in higher plants [8]. Additionally, the xanthonic core is a target of numerous structural modifications, leading to the synthesis of new derivatives [9–12]. The natural and synthetic xanthones together constitute a group of more than 500 compounds. The structural diversity of xanthones is accompanied by their pleiotropic biological activity, which makes them potentially valuable, new therapeutic agents for the treatment of multiple disorders. The most significant activities of xanthones include anticancer [10, 11, 13], antibacterial, and antifungal activities [12]. Xanthones have significant effects in cardiovascular [14] and central nervous system diseases [15]; they also have immunomodulatory and anti-inflammatory potential [16, 17].

Although studies reporting the anticancer properties of xanthone derivatives have been published in recent years, their molecular mechanisms are not fully elucidated. To date, the most widely studied anticancer xanthones are gambogic acid (GA), α-mangostin (MAG), and a synthetic derivative known as 5, 6-dimethylxanthenone-4-acetic acid (DMXAA). One of the most important factors contributing to xanthone pleiotropy is their effect on redox balance inside the cells. Unfortunately, reference data are often contradictory. On one hand, biologically active xanthones have been shown to have strong ROS-stimulating activities [18, 19]; on the other hand, the data also show that some compounds act as antioxidants [20]. The intensively studied GA and MAG have contrary activities, although they both display significant antitumor effects. While ROS-mediated signaling has great importance, especially in cancer and cancer therapy, involvement of ROS and oxidative stress in cells treated with xanthones have not been sufficiently studied. Therefore, the aim of our study was to investigate the role of ROS in the anticancer activity of new aminoalkanol derivatives of xanthone. These compounds were prescreened for their antiproliferative, cytotoxic, and antimigratory effects on different cancer cell lines; the results of this analysis have been published previously [21]. In this study, we analyzed ROS, antioxidants, antioxidant enzymes, mitochondrial potential and cellular senescence under xanthone treatment to establish the relationships between proapoptotic potential and oxidative features of aminoalkanol xanthone derivatives.

## Materials and methods

### Chemicals

DMSO (dimethyl sulfoxide), doxorubicin hydrochloride (DOX), doxycycline hydrochloride, GA, H_2_DCFDA (2′,7′-dichlorodihydrofluorescein diacetate), 30% hydrogen peroxide solution, MAG, N-acetyl-L-cysteine (NAC), and X-gal were purchased from Sigma-Aldrich. Bleomycin sulfate (BLEO) from *Streptomyces verticillus* was purchased from Euro Nippon Kayaku GMBH. Bradford Reagent was purchased from Fermentas. Rhodamine 123 hydrochloride (Rh123), as well as media and chemicals used for in vitro cell cultivation, were purchased from ThermoFisher Scientific.

### Synthesis and purification of aminoalkanol xanthone derivatives

The detailed methodology of synthesis, purification and physicochemical properties of aminoalkanol derivatives of xanthone have been described in our previous paper [21]. All compounds used in the study are summarized in Table 1.

**Table 1:**
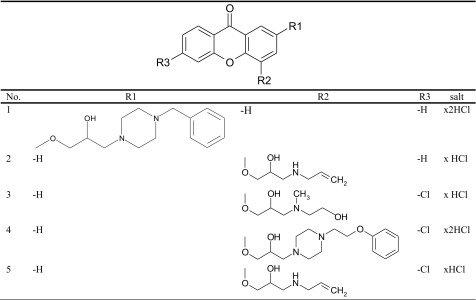
Chemical structure of aminoalkanol xanthone derivatives evaluated in the study

### Cell culture and treatment conditions

C3H/10 T1/2 cells (mouse fibroblasts), genetically modified mouse fibroblasts (OFF-SOD), and the human cancer cell lines A549 and T24 were routinely propagated in suitable media supplemented with 10% FBS and gentamicin at 37 °C in 5% CO_2_. OFF-SOD fibroblasts with Tet-Off-regulated MnSOD expression were generated as described previously [22]. Xanthones were dissolved either in water (synthetic derivatives) or in DMSO (GA and MAG) and stored at −20 °C. Directly before analysis, stock solutions were diluted to obtain the working concentrations. All treatments were performed under non-cytotoxic conditions. IC_50_ values for all xanthones were established in our previous paper [21]. Treatments with all tested compounds were performed for 12 h, except for hydrogen peroxide, which was used in a 6-h treatment. Each compound was investigated in three independent experiments; each experiment was carried out in triplicate.

### ROS detection by H_2_DCFDA

Intracellular ROS were measured based on the detection of the fluorescent product DCF (2′,7′-dichlorofluorescein) yielded from the oxidation of H_2_DCFDA. Cell cultures were incubated in medium containing 10 μM of H_2_DCFDA for 1 h. After the incubation medium was removed, cells were washed with D-PBS. Stained cultures were analyzed for green fluorescence under an inverted fluorescence microscope. In each culture well, 10 vision fields were photographed and analyzed (at least 100 cells in each well). The fluorescence intensity was measured by NIS-Elements AR 3.2 software.

### Total antioxidant status assay

Intracellular level of antioxidants was measured in cell extracts prepared by homogenization in assay buffer using a commercial Antioxidant Assay Kit (Sigma-Aldrich). Concentration of antioxidants was calculated using the standard curves of the absorbance values (λ = 570 nm) generated for Trolox™ dilutions.

### RNA extraction

Total RNA was extracted by the phenol-chloroform method using TRI-Reagent (Sigma-Aldrich) according to the manufacturer’s protocol. RNA extracts were treated with DNase I and purified using a commercial kit (Direct-zol RNA MiniPrep, Zymo Research) to avoid genomic DNA contamination. RNA concentration was determined spectrophotometrically (λ = 260 nm).

### Real-time RT-PCR

Transcript levels of the studied genes, *CAT*, *GPX*, and *SOD1*, were determined by SYBR Green Real-Time RT-PCR assays. Expression of the studied genes was normalized to the endogenous control (*GAPDH* mRNA) by the ∆∆Ct method with the use of the control (untreated) cultures as calibrators. One-step Real-Time RT-PCR assays were carried out using the Mx3000P thermal cycler (Stratagene). Reaction mixtures contained 12.5 μl of 2× Brilliant II SYBR Green RT-PCR Master Mix, 1 μl of reverse transcriptase, 0.3 μM of each sense and antisense primer, 0.1 μg of unknown RNA template, and water to a total volume of 25 μl. All reagents were purchased from Stratagene. The thermal profile was 50°C for 30 min (reverse transcription), 95°C for 10 min, 40 two-step cycles of 94°C for 15 s and 58°C for 30 s, and 72°C for 10 min (real-time PCR), followed by a dissociation protocol (60–95°C; 30 min). The following oligonucleotide sequences were used: *GAPDH* – sense: 5’GAA GGT GAA GGT CGG AGT C3’; antisense: 5’GAA GAT GGT GAT GGG ATT TC3’ (amplification product: 225 bp); *CAT* – sense: 5’TCA GGC AGA AAC TTT TCC ATT T3’; antisense: 5’TGG GTC GAA GGC TAT CTG TT3’ (amplification product: 148 bp [23]); *GPX* – sense: 5’CGG GAC TAC ACC CAG ATG AA3’; antisense: 5’TCT CTT CGT TCT TGG CGT TC3’ (amplification product: 115 bp [23]); *SOD1* – sense: 5’GAA GGT GTG GGG AAG CAT TA3’; antisense: 5’CCA CCG TGT TTT CTG GAT AGA3’ (amplification product: 132 bp [23]).

### CAT, GPX, and SOD activities

Activities of antioxidant enzymes were determined in cell lysates obtained by repeated cell freezing-thawing (2 cycles) and homogenization in extraction buffer (pH = 7.2). Cell lysates were centrifuged (600 g, 10 min), and the resulting supernatants were immediately frozen at −80 °C for further analyses. For GPX activity, cell lysates were prepared in the commercial assay buffer provided by the manufacturer. Catalase activity was determined by kinetic measurement (absorbance at λ = 240 nm) of the decomposition of hydrogen peroxide (30 mM) within 1 min. The enzyme activity was calculated as mU/mg of protein. Protein concentration was evaluated by Bradford assay. Activity of GPX was determined spectrophotometrically (λ = 340 nm) using the commercial Glutathione Peroxidase Assay Kit (Abcam). Activity values (nM of NADPH per 10^6^ cells) were calculated from a standard curve generated for NADPH standards. SOD activity was evaluated spectrophotometrically (λ = 450 nm) by a commercial SOD Determination Kit (Sigma-Aldrich). SOD standards were used to obtain a standard curve for absolute activity assessment. The enzyme activity was calculated as units per milliliter (U/ml).

### Analysis of mitochondria by Rh123 staining

Changes in mitochondrial membrane potential were estimated by Rh123 staining according to earlier protocols [24] with minor modifications. After the treatments, cell cultures were washed with D-PBS and incubated in medium containing 10 μM Rh123 for 30 min. Then, cells were washed with D-PBS and incubated for 1 h in fresh medium to allow efflux of excess dye. After 1 h, cells were again washed with D-PBS, and fluorescence was measured using a 492/521 nm filter. Additionally, we analyzed Rh123-stained mitochondria microscopically to investigate their morphology followed by xanthone treatment. Cells were seeded on sterile microscopic coverslips incubated in culture dishes and analyzed under a fluorescence inverted microscope using an immersion objective (magnif. 1000×). In each culture, 10 vision fields were photographed and analyzed, which was equal to approximately 70 cells.

### Apoptosis detection

Direct detection of apoptotic cells was performed by microscopic analysis. Cells were washed with D-PBS and fixed directly on culture plates for 10 min. Then, cells were stained with annexin V Cy3 conjugate and 6-carboxyfluorescein diacetate (6-CFDA) using a commercial kit (Sigma-Aldrich). All analyses were carried out under an inverted fluorescence microscope. In each culture well, 10 vision fields were photographed and analyzed (at least 100 cells in each well).

### Senescence-associated β-galactosidase activity (SA-β-gal)

For evaluation of the influence of the studied compounds on cellular senescence, β-galactosidase activity assays were performed according to Debacq-Cheniaux et al. [25]. Cells were cultivated for 5 days. During cultivation, the treatment with the studied compounds was performed thrice (days: 1–3-5). After five days, cells were washed with D-PBS, fixed, and stained with X-gal (1 mg/ml) overnight at 37 °C. Microscopic analyses to evaluate the number of β–gal-positive cells were performed with an inverted microscope. At least 300 cells were analyzed in each well.

### Statistical analysis

Quantitative data were compared by Student’s t test or Mann-Whitney U test. For multiple comparisons, ANOVA or ANOVA Kruskal-Wallis were used; *p* < 0.05 was considered significant. All calculations were performed with Statistica v. 10.0 software.

## Results

### Xanthones induce oxidative stress, which can be partly reversed by NAC

**Fig. 1:**
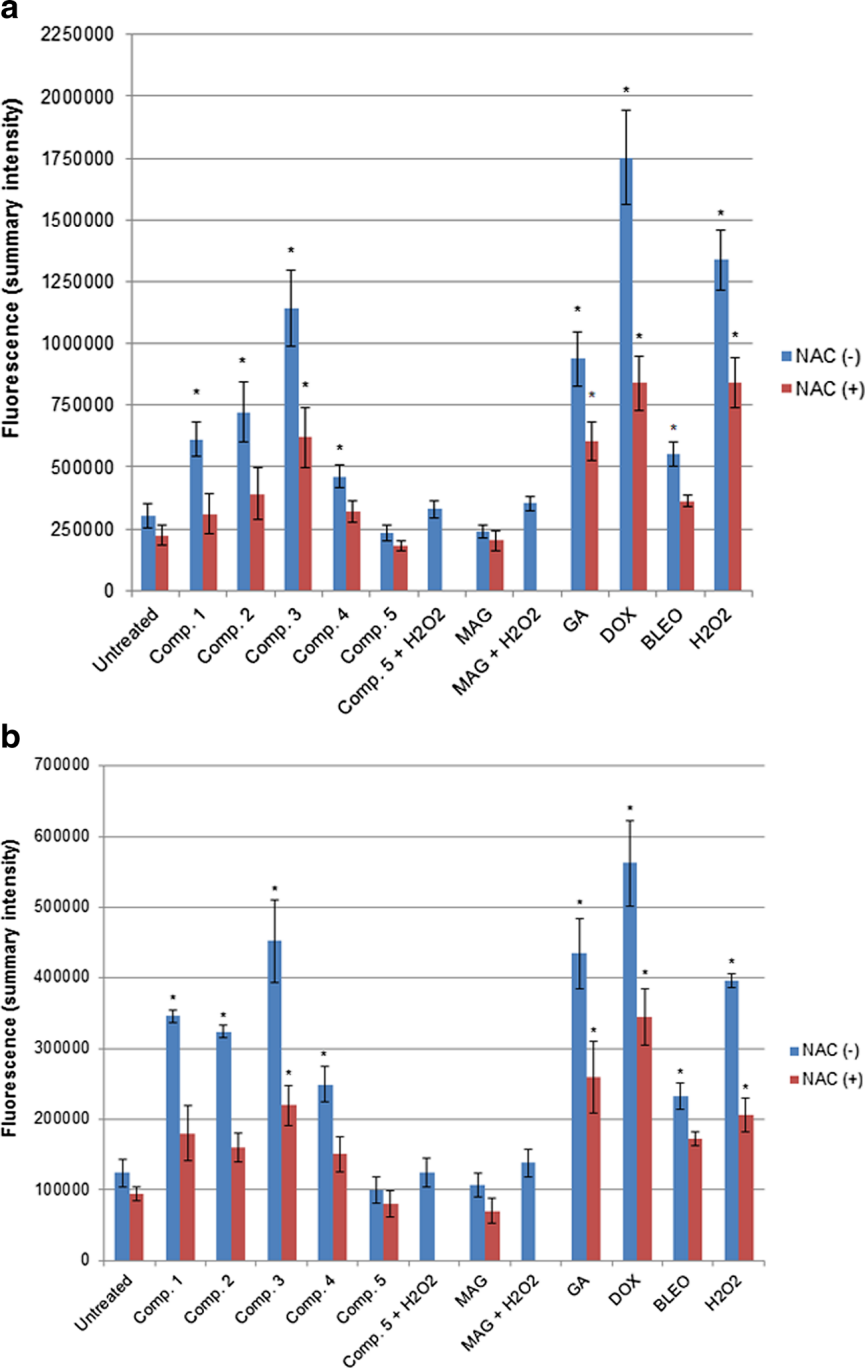
Results of ROS detection by H_2_DCFDA in A549 (**a**) and T24 (**b**) cell cultures. NAC was used in each analysis as the control antioxidant. For each culture, 10 vision fields of three independent experiments were captured and analyzed. Mean value (+/− stand. Dev.) of the fluorescence (summary intensity) was measured by NIS-Elements AR 3.2 software. * depicts statistically significant difference vs. untreated controls (*p* < 0.05)

Oxidative stress was evaluated by the measurement of intracellular levels of ROS and antioxidants. ROS levels were estimated by measuring the DCF (2′,7′-dichlorofluorescein) fluorescence intensity. This method is commonly used in ROS investigations and is based on the application of H_2_DCFDA (acetylated form of DCF), which is consecutively deacetylated inside the cells by intracellular esterases. The resulting molecule is oxidized by intracellular ROS to produce a fluorescent product, DCF. Intracellular levels of antioxidants were measured spectrophotometrically in cell extracts using a commercial antioxidant assay kit based on the concentration-dependent suppression of ferryl myoglobin radicals by intracellular antioxidants. To evaluate the relative strength of the studied xanthones we performed the same analysis for well-established ROS inducers (hydrogen peroxide and chemotherapeutic drugs: BLEO and DOX) and an ROS scavenger, NAC. Statistical analysis revealed the significant increase in ROS levels in A549 and T24 cell cultures after treatments with our synthetic aminoalkanol xanthones (compounds 1–4), and with GA (the natural xanthone) (Fig. 1). The induction of ROS was equally high for GA and compound 3, and it was very similar to that of DOX, the strongest ROS inducer used in the study, and exceeded the activity of BLEO. The overall ROS levels were higher in A549 cell cultures than in T24 cultures (*p* = 0.008), suggesting that this cell line had a higher degree of the intrinsic oxidative stress.

**Fig. 2:**
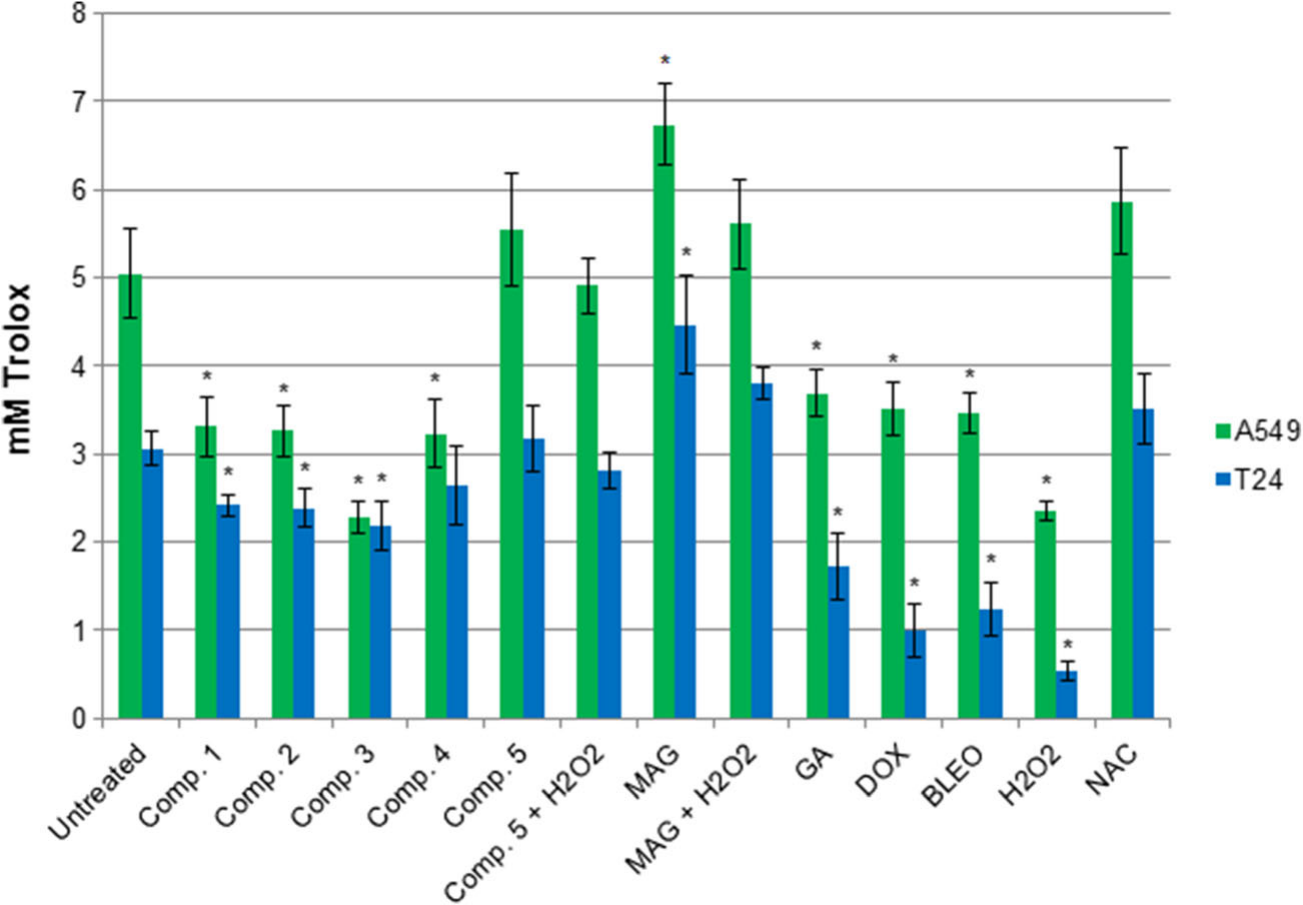
Total antioxidant concentrations in cell lysates of A549 and T24 cultures treated with the studied compounds. Concentration of antioxidants were calculated using the standard curves of the absorbance values of standards (Trolox dilutions). NAC was used in each analysis as the control antioxidant. Graph presents the mean value (+/− stand. Dev.) of three independent experiments, each in triplicate. * depicts statistically significant differences vs. untreated controls (*p* < 0.05)

Simultaneous treatment with the studied compounds and NAC led to significant decrease in ROS levels (Fig. 1). NAC displayed its ROS-scavenging activity primarily in cultures where ROS levels were significantly increased by oxidative compounds, while in untreated controls or cultures treated with MAG or compound 5, NAC resulted in a smaller decrease in ROS levels. Thus, we found that the antioxidant NAC partly reversed the oxidative activity of the studied compounds. Analysis of the total antioxidant pools in the cellular extracts confirmed the results of ROS examinations: strong ROS-inducers resulted in lower concentrations of antioxidants (Fig. 2). The most potent decrease in antioxidant levels was generated by hydrogen peroxide. This assay also revealed that compound 5, MAG, and NAC contributed to the total antioxidant potential of the treated cultures.

### Xanthone-induced oxidative stress influences the expression of CAT, GPX, and SOD

**Fig. 3:**
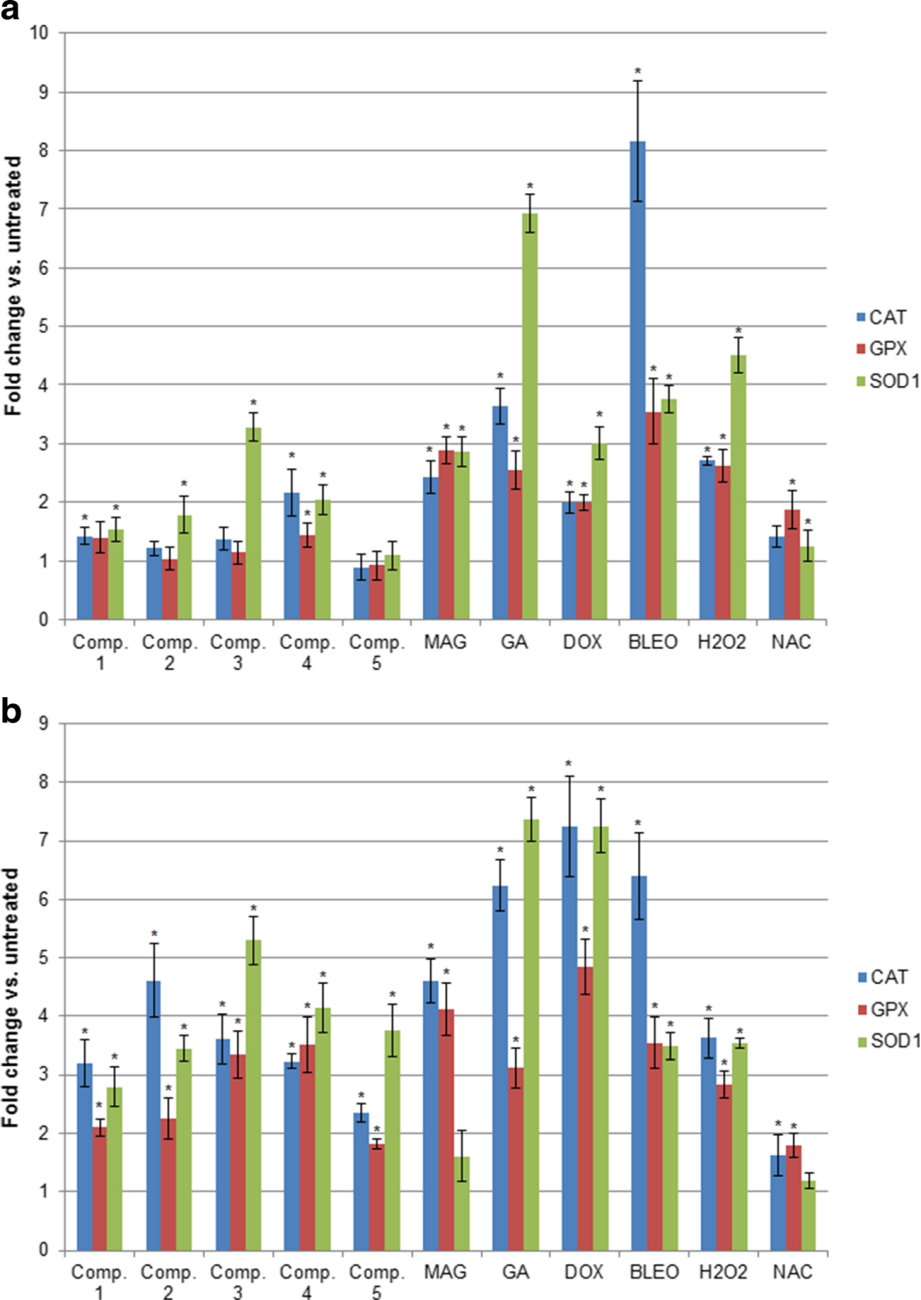
Relative mRNA levels of the genes encoding antioxidant enzymes in A549 (**a**) and T24 (**b**) cancer cell cultures treated with the studied compounds (mean +/− stand. Dev.). mRNA levels of each gene were measured by real-time RT-PCR. Relative expression was calculated by the ∆∆Ct method and expressed as the mean fold change of mRNA expression compared to a calibrator (untreated control). * depicts statistically significant differences vs. untreated controls (*p* < 0.05)

Oxidative stress induced by the xanthone treatment was also confirmed by the increased mRNA expression of the genes encoding antioxidant enzymes, such as *CAT*, *GPX*, and *SOD1* (Fig. 3). The most significant increase was observed in *SOD1* expression, which was enhanced in both cell lines by all the synthetic xanthones (except compound 5) as well as GA and reference compounds (DOX, BLEO and hydrogen peroxide). MAG also stimulated *SOD1* expression but only in A549 cultures, while the reference antioxidant NAC did not influence *SOD1* mRNA levels. *CAT* and *GPX* levels were also affected by the xanthone treatment, and significant differences were observed in both cell lines. Altogether, *CAT* and *GPX* expression levels increased significantly following treatment with all synthetic xanthones in T24 cultures, while in A549 cells, the changes in expression were less dramatic. GA, MAG, and the reference compounds similarly induced *CAT* and *GPX* mRNA levels in both cell lines (NAC increased only *GPX* expression in both cell lines). These results suggest that expression of antioxidant enzymes at the transcriptional level is very sensitive to treatment with anticancer agents and that both pro- and antioxidant compounds may stimulate their expression.

**Fig. 4:**
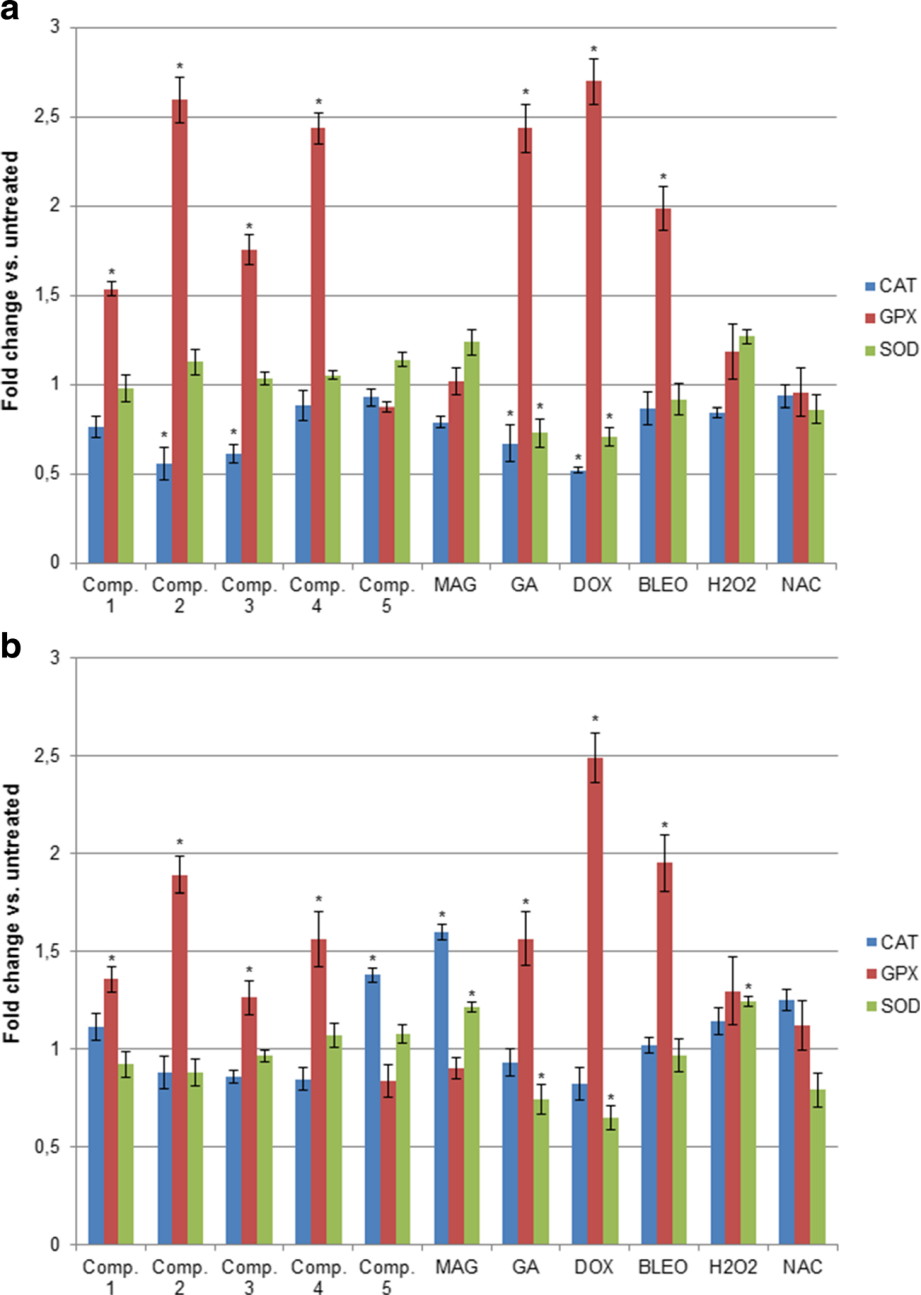
Activity of the antioxidant enzymes measured in cell lysates of A549 (**a**) and T24 (**b**) cultures (mean +/− stand. Dev.). All measurements were performed using spectrophotometric methods described in the *Materials and Methods* section. Graph presents the mean value (+/− stand. Dev.) of fold change compared to control (untreated) cultures. * depicts statistically significant differences vs. untreated controls (*p* < 0.05)

In contrast to mRNA expression, activity of the studied enzymes did not change significantly following treatment in most cases. CAT activity in T24 cell cultures was very similar in all samples except for cells treated with MAG and compound 5, where significant increases were observed. In A549 cell cultures, CAT activity decreased significantly in samples treated with compounds 2 and 3, GA, and DOX. The overall activity of CAT was significantly higher in T24 than in A549 cells (Fig. 4). GPX activity increased significantly in cultures treated with most compounds, and this enzyme showed the most significant changes among all three enzymes analyzed in our study. The highest enhancement was observed for compounds 2 and 4, GA, DOX, and BLEO compared to the control cultures (Fig. 4). Total SOD activity measurements revealed that statistically significant changes were observed only in cultures treated with the natural xanthones: MAG (only in T24), GA, and DOX, as well as T24 cultures treated with hydrogen peroxide, where the highest increase was observed (Fig. 4). As in other assays, MAG and GA exerted completely opposite effects, leading to significant increases and decreases in SOD activity, respectively. These results indicate that MAG was the only compound in the analysis that significantly contributed to the improvement of the antioxidant cellular defense system, while the strongest oxidative compounds, GA, and DOX, led to significant depletion of cellular pools of active SOD.

### Mitochondrial dysfunction and apoptosis enhancement correspond with xanthone-induced oxidative stress

**Fig. 5:**
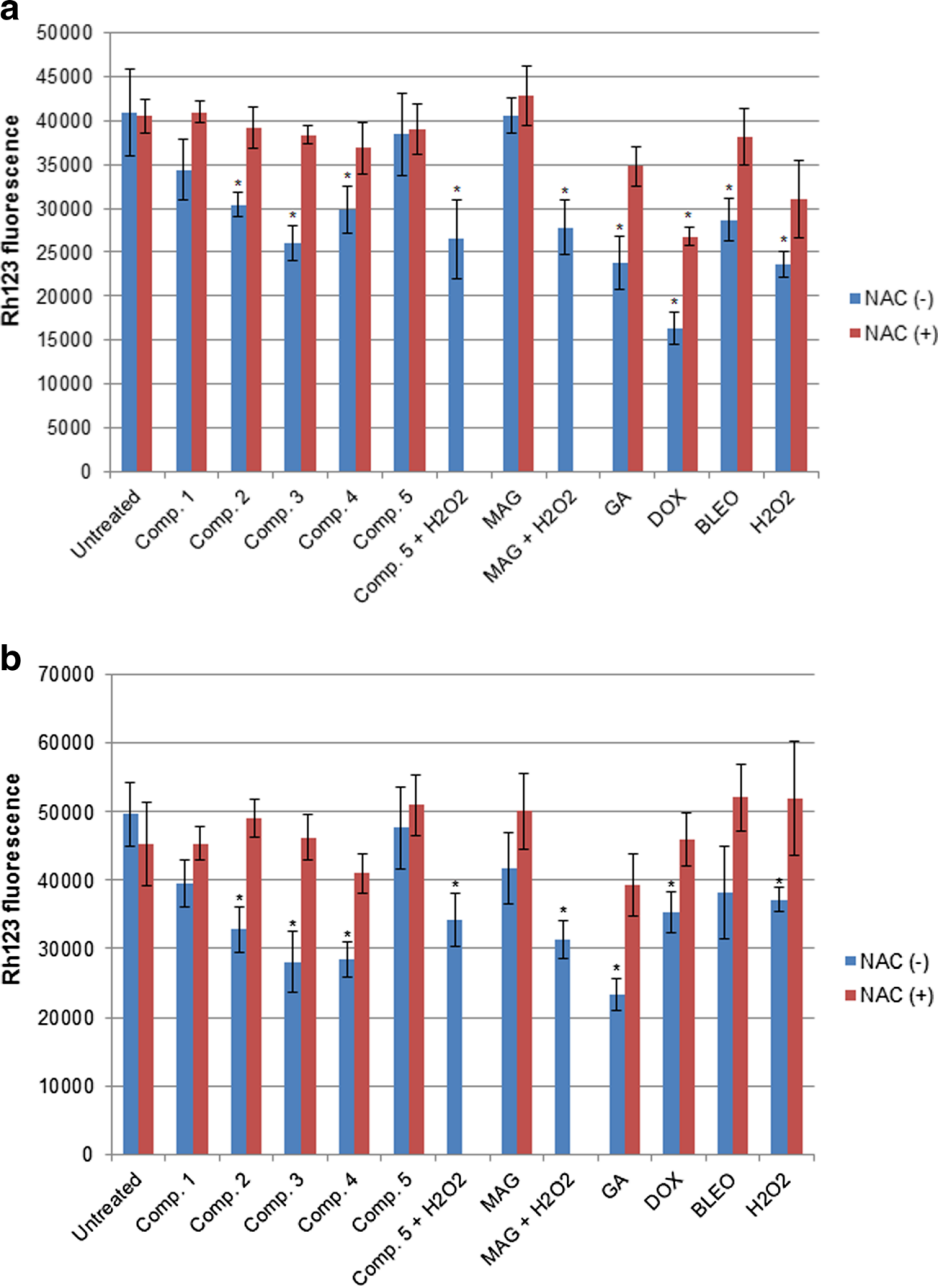

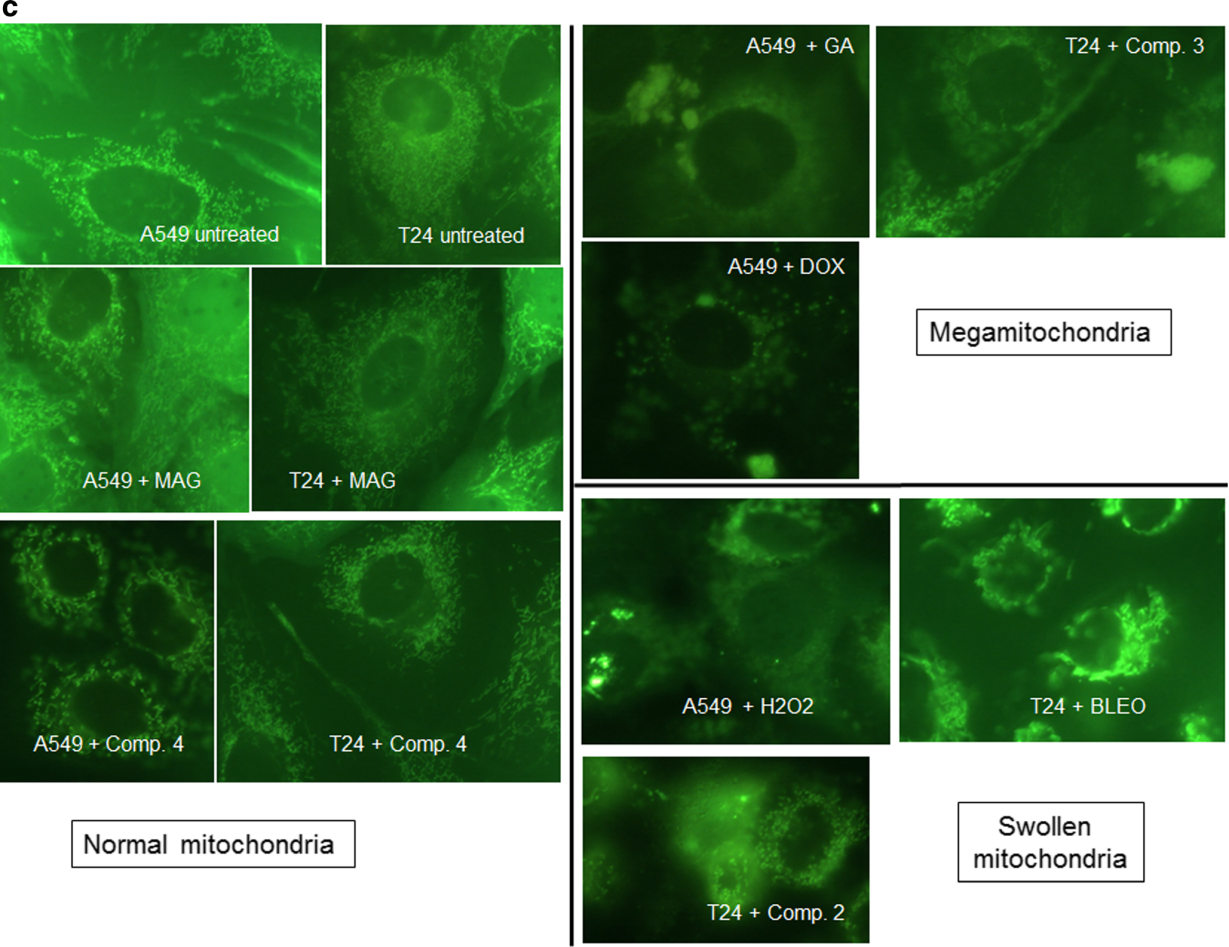
Evaluation of mitochondria using Rh123 staining. Results of Rh123 fluorescence measurements (mean +/− stand. Dev.) in A549 (**a**) and T24 (**b**) cells indicating the loss of mitochondrial membrane potential under xanthone treatment. * depicts statistically significant differences vs. untreated controls (*p* < 0.05). NAC was used in each analysis as the control antioxidant. **c** Representative images of A549 and T24 cells stained with Rh123. The symptoms of mitochondrial swelling can be observed as an increase in size (<3 times), and megamitochondria are the large structures (increased by more than thrice), which are usually accompanied by an overall decrease in Rh123 fluorescence and diffusion of Rh123 to the cytoplasm indicating the general deterioration of mitochondrial function

To investigate the influence of xanthone treatment on cellular mitochondria, we analyzed mitochondrial morphology and transmembrane potential utilizing Rh123 staining. Fig. 5a and b present the results of Rh123 fluorescence measurement in cell cultures subjected to treatment with the studied compounds. These results indicate that all compounds displaying oxidative properties (compounds 1–4, GA, DOX, BLEO, and hydrogen peroxide) decreased the Rh123 green fluorescence values. Depolarization of the mitochondrial membrane is manifested by the loss of Rh123 by mitochondria. The dye is then removed by cellular membrane transporters, which leads to the decrease in intracellular fluorescence intensity. Oxidative stress results in mitochondrial membrane permeabilization. Therefore, Rh123, which normally penetrates mitochondrial membranes, leading to their bright green fluorescence, loses its affinity to mitochondria. The Rh123-stained cells were also examined microscopically to assess the symptoms of mitochondrial dysfunction (Table 2). According to a previous study [26], the appearance of swollen mitochondria or megamitochondria, which are enlarged structures (approximately three times bigger than the normal mitochondria), is a morphological symptom of mitochondrial deterioration. Although mitochondrial morphology was estimated qualitatively, treatment with compounds displaying oxidative potential affected the mitochondrial morphology (Fig. 5c).

**Table 2:** Qualitative results of microscopic evaluation of mitochondrial impairment in A549 and T24 cell cultures treated with the studied compounds

	Swollen mitochondria		Megamitochondria	
	- NAC	+ NAC	- NAC	+ NAC
Untreated	–	–	–	–
Comp. 1	+	–	–	–
Comp. 2	++	–	+	–
Comp. 3	++	+	++	+
Comp. 4	+	–	+	–
Comp. 5	–	–	–	–
Comp. 5 + H_2_O_2_	+	X	+	X
MAG	–	–	–	–
MAG + H_2_O_2_	+	X	+	X
GA	+++	++	+++	+
DOX	+++	++	+++	++
BLEO	++	–	+	–
Hydrogen peroxide	++	+	+++	++

NAC was used in each analysis as the control antioxidant. Mitochondria were stained with Rh123 and analyzed under a fluorescence microscope (magnif. 1000×). For each culture, 10 visual fields of three independent experiments were captured and analyzed. Representative images are presented in Fig. 5a

**Fig. 6:**
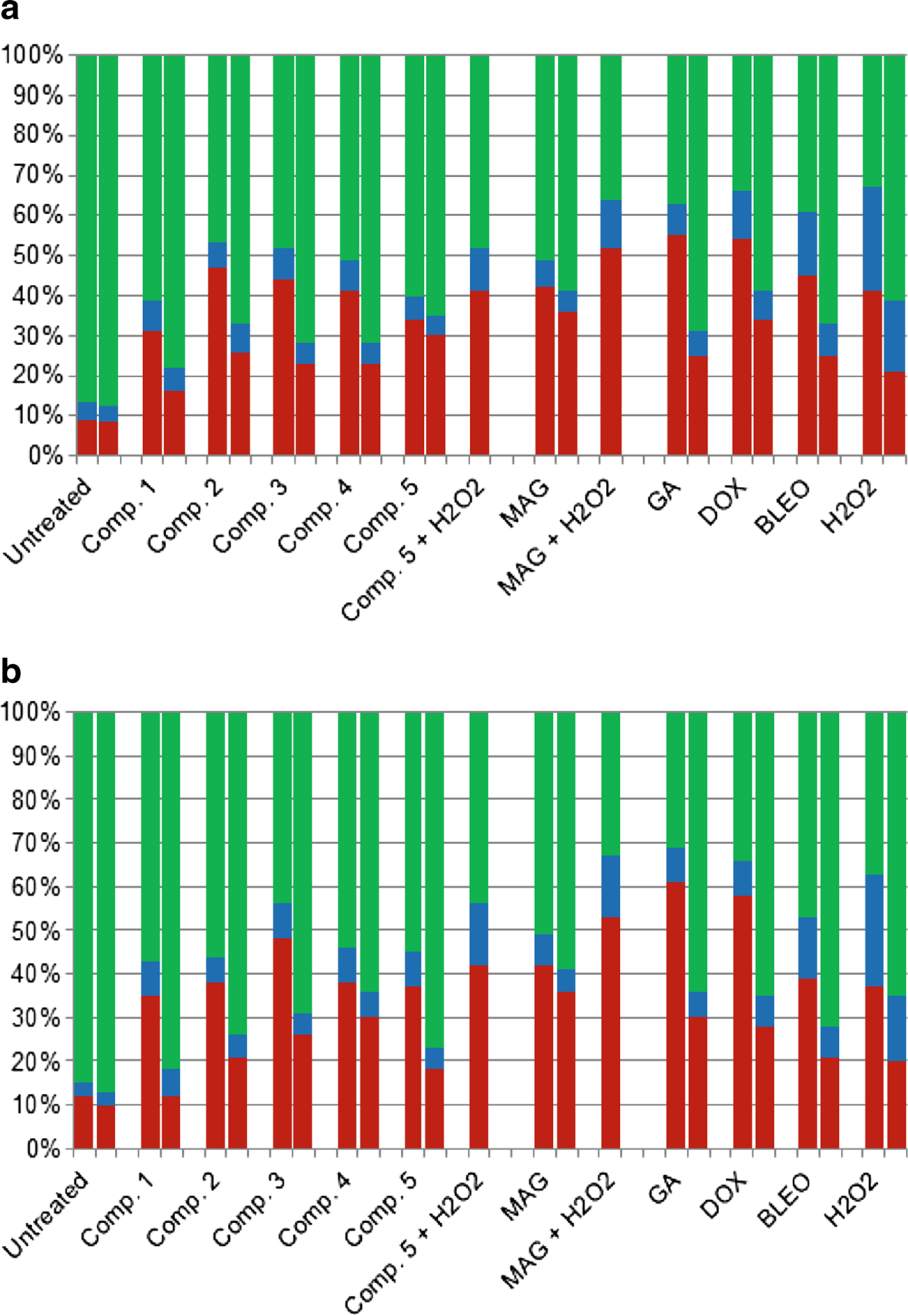
Results of microscopic evaluation of apoptosis and necrosis in A549 (**a**) and T24 (**b**) cell cultures using double staining with annexin V Cy 3-conjugate and 6-CFDA. Viable cells were stained green, cells in the early stages of apoptosis were stained both red and green, and necrotic/dead cells were stained red only. Each column represents distribution of apoptotic, necrotic, and live cell pools (the mean of three independent experiments). NAC was used in each analysis as the control antioxidant

Since mitochondrial dysfunction is an early symptom of apoptosis, we further examined if our studied compounds efficiently induced ROS-mediated cell death using microscopic evaluation of apoptotic, necrotic, and viable cells in the cultures. All compounds used in the study efficiently enhanced apoptosis, as indicated by the elevated number of apoptotic cells in drug-treated cultures. Importantly, MAG and compound 5 also induced apoptosis despite their antioxidant properties. Addition of the antioxidant NAC restored the mitochondrial potential in cell cultures treated with oxidative compounds, such as synthetic xanthones 1–4, GA, and standard compounds (DOX, BLEO, and hydrogen peroxide) (Fig. 5a and b); it also decreased the number of dead cells (Fig. 6). In contrast, NAC did not significantly influence apoptosis induced by MAG or compound 5, and these compounds did not impair mitochondrial activity (Fig. 5). Altogether, these results suggest that cell death is efficiently induced by all the xanthones analyzed in our study, including the antioxidant MAG and compound 5. Although apoptosis was enhanced to a similar degree by antioxidant and pro-oxidant compounds, a difference was observed when NAC was added: it significantly decreased cell death induction only in cultures treated with pro-oxidant compounds. Thus, our results indicate that there is a relationship among ROS stimulation by oxidative xanthones (GA and four aminoalkanol derivatives), mitochondrial impairment, and their pro-apoptotic activities. Addition of NAC reversed the oxidative activity and subsequently led to a decrease in the pro-apoptotic potential only for those compounds that induced oxidative stress in cancer cells.

### Overexpression of MnSOD diminishes xanthone-induced senescence of fibroblasts

**Fig. 7:**
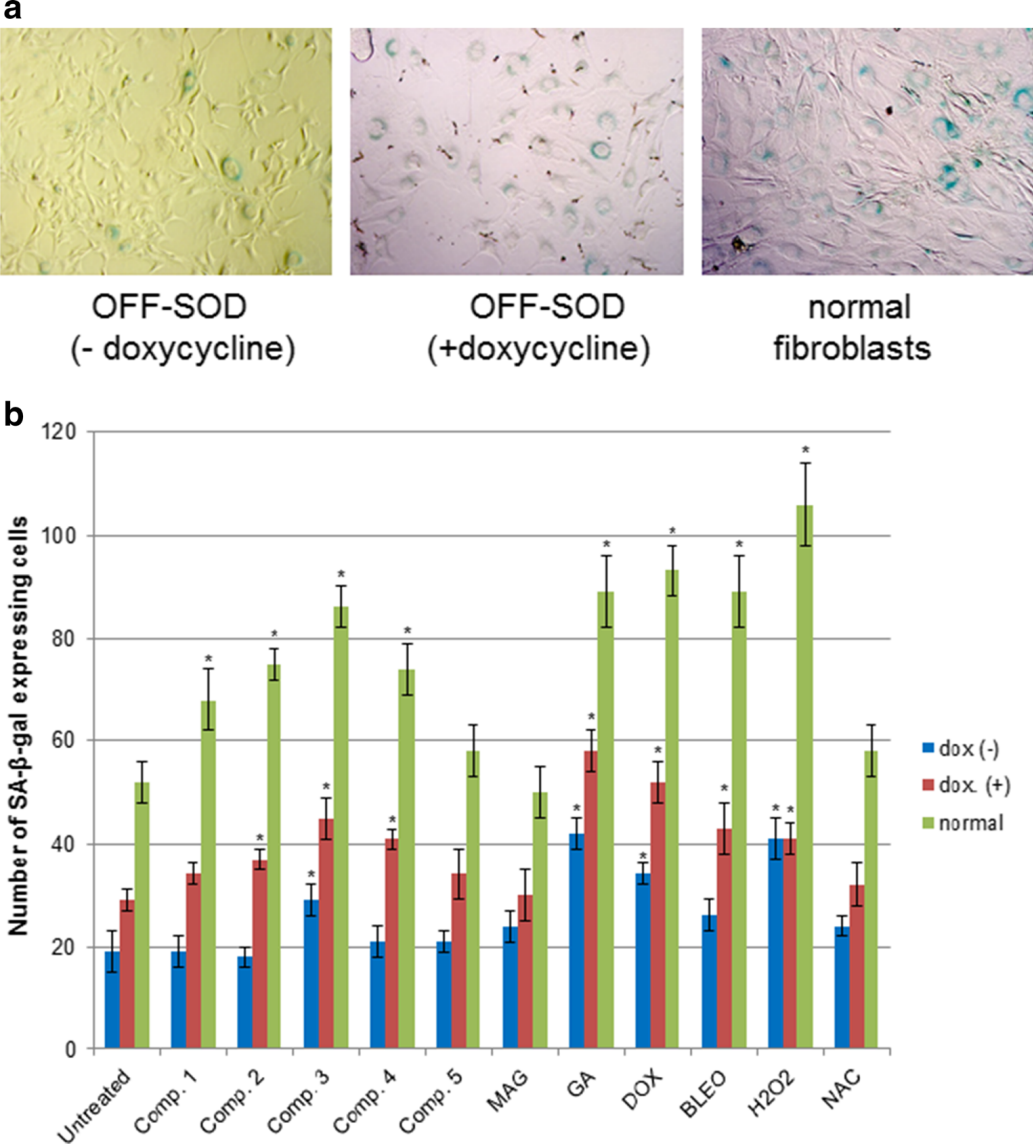
Senescence-induced β-galactosidase expression in genetically modified (OFF-SOD) and normal fibroblasts (C3H/10 T1/2) submitted to drug-induced senescence with the studied compounds. The OFF-SOD with tetracycline-regulated overexpression of MnSOD (Tet-Off system) cell line was established as described in the *Materials and Methods* section. **a** Representative images of cell cultures stained for β-galactosidase activity (magnif. 200×). Cells expressing endogenous β-galactosidase were stained blue-green, while non-expressing cells remained unstained. **b** Quantitative results of microscopic evaluation (mean +/− stand. Dev.). * depicts statistically significant difference vs. untreated controls (*p* < 0.05)

To further elucidate the role of oxygen stress in mediating xanthone activity, we examined an in vitro model of tetracycline-regulated overexpression of an antioxidant enzyme, MnSOD. In this model, we used the OFF-SOD fibroblasts, which overexpress MnSOD under normal conditions. Thus, we established that our studied xanthones induce cellular senescence, and more importantly, we demonstrated that this change was related to the induction of oxidative stress. The results presented in Fig. 7 indicate that overexpression of MnSOD itself protects fibroblasts from the deleterious influence of ROS: β-galactosidase activity was significantly lower in the control (untreated) cultures of OFF-SOD compared to normal (*p* = 0.013) or doxycycline-modulated (*p* = 0.003) cultures. MnSOD also prevented drug-induced senescence in cultures treated with the studied compounds. Only cell cultures treated with compound 3, GA, DOX, and hydrogen peroxide showed a statistically significant increase in β-galactosidase activity. In contrast, normal fibroblast cultures or cultures induced with doxycycline showed increased senescence after the treatment with compounds 1–4, GA, and the control ROS inducers (DOX, BLEO, and hydrogen peroxide). These results indicate that the studied xanthones (GA and compounds 1–4) induce oxidative stress, which leads to cellular senescence. For GA, the activity is so strong that even overexpression of MnSOD cannot eliminate its effect on senescence, which is comparable only to the strongest ROS inducers used in our study: DOX and hydrogen peroxide.

## Discussion

Xanthone derivatives constitute a group of over 500 compounds, which includes numerous compounds with promising anticancer properties. To date, two xanthones, GA and DMXAA, have been qualified in clinical trials [27, 28], and preclinical investigations on many others are being carried out. Elucidation of the molecular mechanism by which xanthones exert their anticancer activity is necessary for further rational screening and synthesis of new derivatives. According to a previous study, the major mechanisms of anticancer activity of xanthones resemble those mechanisms displayed by other well-established chemotherapeutic agents, which primarily include direct interaction with DNA (intercalation, alkylation, cross-linking) and inhibition of topoisomerases [10, 13]. In our previous study, we demonstrated that newly synthesized aminoalkanol xanthone derivatives showed cytotoxic activity and impaired cancer cell motility [21]. In this study, we analyzed the involvement of ROS in the mechanism of anticancer activity of these aminoalkanol xanthones. We compared our compounds with the natural xanthones GA and MAG. Since both of them showed significant antitumor activity, despite their contradictory influence on redox state in cancer cells, we investigated to what extent the involvement of oxidative stress dictates the efficiency of anticancer xanthones. Several anticancer compounds have been shown to stimulate oxidative stress, which contributes to their anticancer mechanism of action. This group includes anthracyclines (e.g., DOX), most alkylating agents and platinum derivatives, bleomycin, mitomycin C, or etoposide. For DOX and BLEO, oxidative stress is an important element of their activity. In the case of xanthones, previous studies have demonstrated that the most active anticancer derivatives act by utilizing oxidative stress [11, 17–19, 29–32]. The results of our present study suggest that most of our synthetic xanthones stimulate oxidative stress in the studied cell cultures. Treatment of A549 and T24 cells with the studied compounds led to significant increases in ROS, decreases in total antioxidant levels, and enhanced expression of antioxidant enzymes at the mRNA level. Oxidative stress induced by these compounds led to mitochondria dysfunction, which correlated with cell death enhancement. It also induced ROS-associated senescence, and overexpression of MnSOD reversed this process. We included in our analysis two natural xanthones, GA and MAG, which are the most extensively investigated anticancer xanthone derivatives. Moreover, they have completely opposite modes of action: GA strongly induces oxidative stress, which is important if not crucial for its anticancer properties [19, 33], while MAG has been demonstrated by numerous studies to be an antioxidant despite its significant anticancer activity. Our results supported those reports. These data, both from previous studies and our analysis, clearly demonstrate that the xanthonic core, which gives rise to a few hundred natural or synthetic derivatives, does not determine xanthone’s activity. Instead, the nature and localization of side substituents dictates the overall biological features of a specific xanthone derivative.

The literature indicates that most anticancer xanthones stimulate ROS, and this significantly contributes to their mode of action. One of the most studied xanthones, GA, has been shown to stimulate ROS in many cancer cell lines [19, 33]. Moreover, GA-mediated ROS generation is responsible for enhancement of apoptosis, and the addition of antioxidants diminished the anticancer activity of GA. Nie et al. confirmed that ROS accumulation generated by GA contributed to mitochondrial damage, which manifested as the loss of transmembrane potential and subsequently the induction of the mitochondrial pathway of apoptosis; NAC addition partly reversed the anticancer activity of GA [19]. Similar observations were confirmed for other, less extensively studied xanthones, either natural or synthetic [29], such as GA-analogues (GNA, cluvenone) [18, 32], EPOX [34], 1-hydroxyl-3-aminoalkoxy derivatives [11] or griffipavixanthone [30]. Finally, ROS have been shown to be the chief mediators of DMXAA activity as an inducer of cytokine secretion, which is an important molecular mechanism of the indirect anticancer and immune modulatory actions of this compound [17].

In our study, we also analyzed the expression and activity of the antioxidant enzymes CAT, GPX, and SOD. While the mRNA expression of all three genes significantly increased in most cultures treated with xanthones, their enzymatic activities were not always changed: the highest changes were observed in cultures treated with the most pro-oxidant compounds. Previous data indicate that therapies based on oxidative agents decrease the activity of antioxidant enzymes due to their consumption in ROS-scavenging reactions [35]. An increase in ROS levels is a signal to enhance the expression of antioxidant enzymes; however, high levels of ROS lead to the depletion of active enzyme pools, and thus, decreased activity may be observed, although their expression is enhanced. Similar observations have been published in other studies analyzing MAG [36, 37] or natural xanthones from *Swertia chirayita* [38]. Other xanthones lack suitable data.

Although most xanthone derivatives described in the references have pro-oxidant features, MAG, one of the most active natural xanthones, as well as other natural xanthones from mangosteen (*Garcinia mangostana*) fruit, have been shown to display antioxidant properties [20, 31, 36, 37, 39]. MAG treatment leads to a dose-dependent inhibition of hypoxia-induced migration of pancreatic cancer cells by diminishing the elevated levels of ROS as efficiently as NAC [40]. Antioxidants such as MAG may serve as adjuvants in therapies utilizing strong pro-oxidant agents to exert cytoprotective activity toward the normal cells in hypoxic conditions where high levels of ROS are observed [41]. However, MAG and other mangosteen extract components have also been shown to stimulate apoptosis in various cancer cell lines, and this effect has been linked with their capacity to induce ROS and destabilize mitochondria (loss of transmembrane potential, swelling, cytochrome c release) [42–44]. Sun et al. reported that MAG scavenged hydroxyl radicals, superoxide anions, and hydrogen peroxide but increased the levels of singlet oxygen, which leads to apoptosis [31]. Overall, these studies demonstrate two important discoveries: first, whether these natural xanthones serve as antioxidants or apoptosis inducers depends on the concentration used. Second, the key role of singlet oxygen in apoptosis induction has been demonstrated, which links MAG and other antioxidant xanthones with their potential use as anticancer agents either alone or in combination with other chemotherapeutic drugs.

Our study focused on verification of novel synthetic xanthone derivatives and their molecular mechanisms involving ROS signaling. We demonstrated that our aminoalkanol xanthone derivatives promoted oxidative stress and thereby induced the death of cancer cells. Thus, we concluded that the mechanism of action of our xanthones is similar to that exerted by GA, one of the most active anticancer xanthones and a potent ROS inducer. The group of analyzed xanthones included derivatives with different side chains, which was reflected by the different degree of ROS stimulation displayed by these compounds; nevertheless, most of them significantly induced oxidative stress in cancer cell cultures. We showed that ROS constitute an important element of the proapoptotic activity of aminoalkanol xanthones, especially compounds 3 and 4, which even exceeded the oxidative potential of GA in some assays used in the study. Data from previous studies indicate that even the antioxidant MAG mediates its cytotoxic activity in cancer cultures through ROS; thus, based on our results and other studies, we conclude that pro-oxidants should be screened for potential use in anticancer therapy.

## Abbreviations

CAT: catalase
6-CFDA: 6-carboxyfluorescein diacetate
BLEO: bleomycin sulfate
DMSO: (dimethyl sulfoxide)
DOX: doxorubicin
FBS: fetal bovine serum
GA: gambogic acid
GAPDH: glyceraldehyde 3-phosphate dehydrogenase
GNA: gambogenic acid
GPX: glutathione peroxidase
H_2_DCFDA: 2′,7′-dichlorodihydrofluorescein diacetate
MAG: α-mangostin
NAC: N-acetyl-L-cysteine
PBS: phosphate-buffered saline
Rh123: rhodamine 123
ROS: reactive oxygen species
SOD: superoxide dismutase
